# The Proteomics of Colorectal Cancer: Identification of a Protein Signature Associated with Prognosis

**DOI:** 10.1371/journal.pone.0027718

**Published:** 2011-11-18

**Authors:** Donna O'Dwyer, Lynda D. Ralton, Aisling O'Shea, Graeme I. Murray

**Affiliations:** Department of Pathology, University of Aberdeen, Aberdeen, United Kingdom; Ottawa Hospital Research Institute, Canada

## Abstract

Colorectal cancer is one of the commonest types of cancer and there is requirement for the identification of prognostic biomarkers. In this study protein expression profiles have been established for colorectal cancer and normal colonic mucosa by proteomics using a combination of two dimensional gel electrophoresis with fresh frozen sections of paired Dukes B colorectal cancer and normal colorectal mucosa (n = 28), gel image analysis and high performance liquid chromatography–tandem mass spectrometry. Hierarchical cluster analysis and principal components analysis showed that the protein expression profiles of colorectal cancer and normal colonic mucosa clustered into distinct patterns of protein expression. Forty-five proteins were identified as showing at least 1.5 times increased expression in colorectal cancer and the identity of these proteins was confirmed by liquid chromatography–tandem mass spectrometry. Fifteen proteins that showed increased expression were validated by immunohistochemistry using a well characterised colorectal cancer tissue microarray containing 515 primary colorectal cancer, 224 lymph node metastasis and 50 normal colonic mucosal samples. The proteins that showed the greatest degree of overexpression in primary colorectal cancer compared with normal colonic mucosa were heat shock protein 60 (p<0.001), S100A9 (p<0.001) and translationally controlled tumour protein (p<0.001). Analysis of proteins individually identified 14-3-3β as a prognostic biomarker (χ^2^ = 6.218, p = 0.013, HR = 0.639, 95%CI 0.448–0.913). Hierarchical cluster analysis identified distinct phenotypes associated with survival and a two-protein signature consisting of 14-3-3β and aldehyde dehydrogenase 1 was identified as showing prognostic significance (χ^2^ = 7.306, p = 0.007, HR = 0.504, 95%CI 0.303–0.838) and that remained independently prognostic (p = 0.01, HR = 0.416, 95%CI 0.208–0.829) in a multivariate model.

## Introduction

In the western world colorectal cancer (CRC) is the third most common type of cancer and the second most common cause of cancer death [1]. Worldwide one million people each year will develop CRC and the incidence of this tumour is increasing [1]. Most cases of CRC are sporadic resulting from the accumulation of somatic genetic aberrations and are associated with a variety of environmental risk factors [1], [2]. The remaining proportion of cases involve a familial genetic component. Numerous genetic aberrations accumulate including the inactivation of the adenomatous polyposis coli tumour suppressor gene and activation of oncogenes such as K-ras, deletion of chromosome 18q and amplification of 20q [1], [3]. Cumulatively these genetic changes afford the tumour anti-apoptotic, pro-angiogenic and proliferative properties. Recently it has been accepted that CRC is a genetically heterogeneous disease and two distinct pathways of carcinogenesis have been identified. Of sporadic CRC, 85% results from chromosomal instability and the remaining 15% from microsatellite instability [3]. Rather than occurring as a linear multistep process, colorectal carcinogenesis is more likely to be the result of the complex interplay between multiple mutational pathways. This may partly explain the clinical heterogeneity of this disease and the great difference seen in outcome between individual patients [2]. This emphasises the clear requirement to have refined methods of classifying and categorising colorectal cancer by identifying and validating appropriate biomarkers.

Molecular biomarkers can be categorised by their ability to aid prevention, promote early detection, establish prognosis and predict response of patient to specific therapies [4], [5]. The discovery of biomarkers will also aid in the understanding of the biological mechanisms underlying disease development and progression. Whilst genomics including epigenomics and transcriptomics have been influential in biomarker discovery, studying genes and gene expression does not accurately reflect the amount of protein expressed in the cell. Additionally proteins undergo many post-translational modifications which can affect their activation, interactions and function within a cell. Proteomics which is the global study of proteins has a key role in the potential identification of tumour associated biomarkers [6], [7]. The relationship between individual tumour biomarkers and colorectal cancer has been extensively investigated and studies have included biomarkers representing genes and proteins involved in many aspects of tumour development and progression including tumour invasion and metastasis, cell cycle regulation, growth factors and apoptosis associated proteins [5], [8]–[26].

In this study we have used comparative proteomic analysis (two dimensional gel electrophoresis, image analysis of gels and mass spectrometry) to identify proteins which are over-expressed in colorectal cancer, compared with morphologically normal colorectal mucosa. Overexpressed proteins have been validated by immunohistochemistry using a large well characterised set of colorectal cancers and a protein signature associated with prognosis identified.

## Methods

### Two dimensional (2D) gel electrophoresis

2D gel electrophoresis was performed using matched pairs of fresh frozen Dukes' B colon cancer and morphologically normal colonic mucosa (caecum and ascending colon, n = 15 and sigmoid colon n = 13) as previously described [20]–[22], [27]. All cases were selected from the Aberdeen colorectal tumour bank and the clinicopathological details of the samples used for proteomics are noted in Table 1. None of patients had received chemotherapy or radiotherapy prior to surgery. On collection, both tumour and normal colorectal mucosa were dissected from colorectal cancer excision specimens within 30 minutes of surgical removal, and immediately frozen in liquid nitrogen and stored at −80°C prior to analysis.

**Table 1:** Clinico-pathological details of tumour samples used for proteomic analysis.

	Percent (number)
Sex	
Male	61% (17)
Female	39% (11)
Age (mean:range)	73: 60–92
<70	36% (10)
≥70	64% (18)
Tumour differentiation	
Well/moderate	93% (26)
Poor	7% (2)
Extramural vascular invasion	
Present	11% (3)
Absent	89% (25)
Tumour site	
Proximal	43% (12)
Distal	57% (14)
Tumour stage	
T3	82% (23)
T4	8% (5)
Nodal stage	
N0	100% (28)
Mean lymph node yield	18 lymph nodes per case
Survival	
Mean	76 months (95% CI 55–97 months)

22 of the tumours used for the proteomics studies are also represented in the colorectal cancer TMA used for protein validation.

Frozen sections (20 microns thickness, n = 30) of each sample were cut and solubilised in lysis buffer [27]. One section (10 microns thickness) from each sample was stained with haematoxylin and eosin and the morphological diagnosis confirmed by light microscopic examination. Following solubulisation, the samples were centrifuged to remove insoluble cellular debris and treated with DNAse. 2D gel electrophoresis was performed in duplicate for each sample using 13 cm pI3-10 non-linear immobilon strips (GE Health Care, Little Chalfont, UK) with proteins being separated according to charge (300 V, 6 mins, 3500 V, 90 min, 3500 V, 300 min), and subsequently molecular weight (100 V, 25 mA per gel for 60 min). Following completion of the electrophoresis, gels were stained with coomassie blue to visualize proteins spots.

### Gel imaging and analysis

The gels were then scanned to produce 256 grey scale 24 bit images which were saved as TIFF files. The imaged gels were analysed using Progenesis SameSpots software (Non-Linear Dynamics, Newcastle-upon-Tyne, UK). All gel images were imported into Progenesis SameSpots for analysis. Image quality assessment was also done using the SameSpots software to ensure all images were in the correct format for analysis and had no other problems that could interfere with subsequent image analysis. All gels were initially automatically aligned onto one reference gel using the analysis software, then manually aligned to ensure proper alignment of all gels, allowing all spots to be detected, normalised and matched on all gels. Artefacts (e.g., dust particles or streaks detected as protein spots) were removed by manual editing. Reference image gels were created following gel alignment using the analysis software. Once aligned, gels were automatically analysed using the Progenesis SameSpots software. Gels were separated into 2 groups as either tumour or normal gels. Statistical analysis of protein expression levels were then determined for each spot based on mean spot volume, and differences in protein expression between tumour and normal gels were assessed by ANOVA. Spots with a p≤0.05 were selected for inclusion in the results. Multivariate analysis was also done using Progenesis SameSpots and both correlation analysis and principle components analysis was performed on the imaged gels. Correlation analysis was performed on log normalised spot expression levels to group spots together according to similarities in their expression profiles. Principal components analysis used spot expression levels across all gels to separate the gels according to expression variation, allowing a graphical representation of the multidimensional data, clustered into the two groups; tumour and normal. A final report showing all analysed spots on the gel together with ANOVA values, ranks and expression profiles for each spot based on the average normalised volume for the groups was then produced.

### Liquid chromatography–tandem mass spectrometry

Following 2D gel electrophoresis and image analysis of the gels the protein spots of interest (those spots which were significantly increased in the tumour samples) were excised from the gels and proteins identified by liquid chromatography-tandem mass spectrometry.

Proteins in the gel pieces were digested with trypsin (sequencing grade, modified; Promega UK, Southampton, UK) using an Investigator ProGest robotic workstation (Genomic Solutions Ltd., Huntingdon, UK). Briefly, proteins were reduced with DTT (60°C, 20 min), S-alkylated with iodoacetamide (25°C, 10 min) then digested with trypsin (37°C, 8 h). The resulting tryptic peptide extract was dried by rotary evaporation (SC110 Speedvac; Savant Instruments, Holbrook, NY, USA) and dissolved in 0.1% formic acid for LC-MS/MS analysis.

Peptide solutions were analysed using an HCTultra PTM Discovery System (Bruker Daltonics Ltd., Coventry, UK) coupled to an UltiMate 3000 LC System (Dionex (UK) Ltd., Camberley, Surrey, UK). Peptides were separated on a monolithic capillary column (200 µm internal diameter ×5 cm in length; Dionex). Eluent A was 3% acetonitrile in water containing 0.05% formic acid, eluent B −80% acetonitrile in water containing 0.04% formic acid with a gradient of 3%–45% B in 12 minutes at a flow rate of 2.5 µL/min. Peptide fragment mass spectra were acquired in data-dependent AutoMS(2) mode with a scan range of 300–1500 m/z, 3 averages, and up to 3 precursor ions selected from the MS scan 100–2200 m/z). Precursors were actively excluded within a 1.0 min window, and all singly charged ions were excluded.

Peptide peaks were detected and deconvoluted automatically using data analysis software (Bruker). Mass lists in the form of Mascot generic files were created automatically and used as the input for Mascot MS/MS Ions searches of the NCBInr database using the Matrix Science web server (www.matrixscience.com). The default search parameters used were: enzyme = trypsin, maximum missed cleavages = 1; fixed modifications = carbamidomethyl (C); variable modifications = oxidation (M); peptide tolerance ±1.5 Da; MS/MS tolerance ±0.5 Da; peptide charge = 2+ and 3+ and instrument = ESI-TRAP.

Both two dimensional gel electrophoresis and mass spectrometry were carried out by the University of Aberdeen Proteome facility (www.abdn.ac.uk/ims/proteomics/).

### Development of colorectal cancer tissue microarray

A colorectal cancer tissue microarray was constructed containing normal colon mucosa (n = 50), primary (n = 515) and metastatic colorectal cancer (n = 224) as previously described [28], [29].

All cases were selected from the Aberdeen colorectal tumour bank. In total, tumour samples from 515 patients were involved in this study, in each case, a diagnosis of primary colorectal cancer had been made, and the patients had undergone elective surgery for primary colorectal cancer, in Aberdeen, between 1994 and 2007. 99 tumours were from the period 1994–1998, 199 tumours were from 1999–2003 and 217 tumours were from the period 2004–2007. None of the patients had received any pre-operative chemotherapy or radiotherapy. The data for the patients and their tumours included in this study is detailed in supporting information Table S1. The mean lymph node yield for all tumours in this study was 13.4 lymph nodes per tumour and for node negative tumours the mean lymph node yield was 14.4 (lymph node yield refers to the total number of lymph nodes retrieved from each colorectal cancer resection specimen). Survival information was available for all patients and at the time of censoring patient outcome data there had been 237 (46%) deaths (all cause mortality). The mean patient survival was 114 months (95% CI 105–122 months). The colorectal cancer excision specimens were received fresh, opened above and when appropriate below the tumour, washed in cold water and then fixed in 10% neutral buffered formalin for at least 48 hours at room temperature and representative blocks were embedded in wax. Sections were stained with haematoxylin and eosin for histopathological diagnosis and the tumours were reported according to The Royal College of Pathologists guidelines which incorporate guidance from TNM5 of the TNM staging system.

A colorectal cancer tissue microarray was constructed containing normal colon mucosa (n = 50), primary (n = 515) and metastatic colorectal cancer (n = 224). The metastases were all from tumour involved lymph nodes of the Dukes C cases. Each normal mucosal sample was acquired from at least 10 cm distant from the tumour as previously described [28], [29]. All the cases were reviewed and areas of tissue to be sampled were first identified and marked on the appropriate haematoxylin and eosin stained slide by an expert consultant gastro-intestinal pathologist (GIM). Two 1 mm cores were taken from these areas of the corresponding wax embedded block using a Beecher Instruments tissue microarrayer (Sun Prairie, WI, USA) and placed in a recipient paraffin block. Following transfer, the recipient array block was heated to 37°C, and a glass slide was used to carefully press down the cores to ensure they were all at the same level within the recipient wax block.

### Immunohistochemistry

Immunohistochemistry for each antibody (Table 2) was performed with the biotin free Dako Envision™ system (Dako, Ely, UK) using a Dako autostainer (Dako) as previously described [28], [30], [31]. Sections of the tissue microarray were dewaxed in xylene, rehydrated in alcohol and an antigen retrieval step performed. This step consisted of microwaving the sections fully immersed in 10 mM citrate buffer at pH6.0 for 20 minutes in an 800 W microwave oven operated at full power. The sections were then allowed to cool to room temperature. The primary antibody appropriately diluted (Table 2) in antibody diluent (Dako) was applied for 60 minutes at room temperature, washed with buffer (Dako) with subsequent peroxidase blocking for 5 minutes (Dako). This was followed by a single 2 minute buffer wash after which pre-diluted peroxidase-polymer labelled goat anti-mouse/rabbit secondary antibody (Envision™, Dako) was applied for 30 minutes at room temperature, followed by further washing with buffer to remove unbound antibody. Sites of peroxidase activity were then demonstrated with diaminobenzidine as the chromogen applied for three successive 5 minute periods. Finally sections were washed in water, lightly counterstained with haematoxylin, dehydrated and mounted. Omitting the primary antibody from the immunohistochemical procedure and replacing it either with antibody diluent or non-immune rabbit serum as appropriate acted as negative controls. Positive controls were tissues known to express the individual protein.

**Table 2:** Details of the antibodies used in this study.

Antibody	Supplier	Antibody type	Reference number (clone number)	Optimal dilution
14-3-3β	Sigma	rabbit polyclonal	HPA011212	1/500
Aldehyde dehydrogenase 1 (ALDH1)	BD Biosciences	mouse monoclonal	611194 (44)	1/1600
Enolase 1(ENO1)	Abcam	rabbit polyclonal	ab85086	1/50
Glyceraldehyde 3-phosphate dehydrogenase (GAPDH)	Abcam	mouse monoclonal	ab75479 (1A10A10)	1/2000
Glutathione peroxidase 1 (GPX1)	Abcam	rabbit polyclonal	ab22604	1/1000
Heat shock protein 60 (HSP60)	Abcam	rabbit polyclonal	ab46798	1/2000
Isocitrate dehydrogenase 1 (IDH1)	Sigma	rabbit polyclonal	HPA035248	1/250
Lactate dehydrogenase B (LDHB)	Abcam	rabbit monoclonal	ab53292 (EP1565Y)	1/800
Major vault protein (MVP)	Abcam	rabbit polyclonal	ab97311	1/400
Nucleophosmin (NPM1)	Sigma	rabbit polyclonal	HPA011384	1/800
Prohibitin (PHB)	Abcam	rabbit monoclonal	ab75771 (EP2804Y)	1/100
Peptidylprolyl isomerase B (PPIB)	Abcam	rabbit polyclonal	ab16045	1/1600
Peroxiredoxin 1 (PRDX1)	Abcam	rabbit polyclonal	ab59538	1/400
S100A9	Abcam	rabbit monoclonal	ab92507 (EPR3555)	1/800
Translationally controlled tumour protein (TCTP)	Abcam	rabbit polyclonal	ab37506	1/4000
MLH1	BD Pharmingen	mouse monoclonal	554073 (G168-728)	1/100
MSH2	Merck	mouse monoclonal	NA27 (FE11)	1/50

The sections were evaluated by light microscopic examination and the intensity of immunostaining in each core assessed independently by two investigator (DO'D and GIM) using a scoring system previously described for the assessment of protein expression in tumour microarrays [28]–[31]. The intensity of immunostaining in each core was scored as negative, weak, moderate or strong. The subcellular localisation (either nuclear or cytoplasmic) of the immunostaining was also assessed. Variation in immunostaining between cores of each case was not identified. Any discrepancies in the assessment of the tissue cores between the two observers were resolved by simultaneous microscopic re-evaluation.

### Assessment of microsatellite instability status

Microsatellite instability status (MSI) was assessed by immunohistochemistry using antibodies to MLH1 and MSH2 (Table 2) as described previously [30].

### Statistics

Statistical analysis of the immunohistochemical data including the Mann-Whitney U test, Wilcoxon signed rank test, chi-squared test, hierarchical cluster analysis, Kaplan-Meier survival analysis, log-rank test and Cox multi-variate analysis (variables entered as categorical variables) including the calculation of hazard ratios and 95% CIs were performed using PASW v18.0.2 for Windows XP™ (SPSS UK, Ltd, Woking, UK). The log rank test was used to determine survival differences between individual groups. A probability value of p≤0.05 was regarded as significant. To explore the influence of different cut-off points in relation to survival the immunohistochemical scores for each marker were dichotomized. The groups that were analysed were negative versus any positive staining, negative and weak staining versus moderate and strong staining and negative, weak and moderate staining versus strong staining. Hierarchical cluster analysis was carried out using the furthest neighbour method with the square Euclidean distance as the cluster measure and cluster analysis was performed without any transformation of the data or imputation of missing values [18], [19].

### Ethics

The project had the approval of The North of Scotland Research Ethics Committee (ref. no. 08/S0801/81). Written informed consent was obtained from participants who provided fresh samples of tissue for the proteomics component of the study. The research ethics committee waived the requirement for written consent for the retrospective tissue samples included in the colorectal cancer tissue microarray.

## Results

### Proteomics

**Figure 1:**
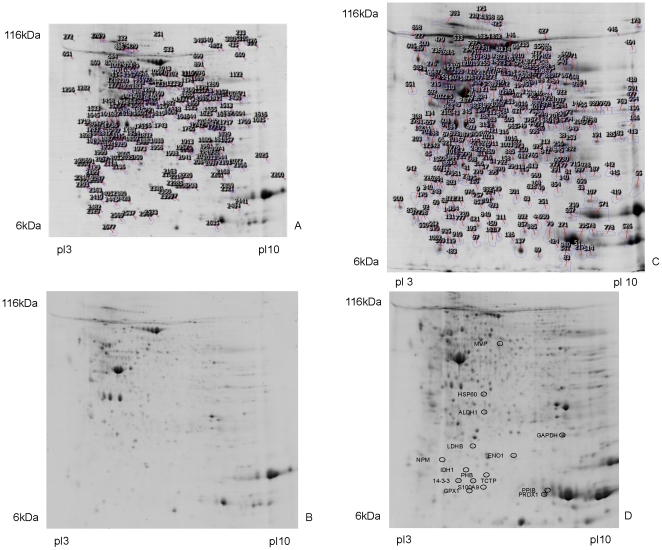
Tumour and normal 2D gels. Representative reference 2D gels of normal colon (A) and colon tumour (C). These are the annotated reference gels created by the Progenesis Same Spots gel image analysis software for the analysis of individual gels. The number of each spot is assigned by the image analysis software. For easier visualisation of individual spots representative non-annotated 2D gels of normal colon and colon tumour are shown in panels B and D respectively. The proteins which were validated by immunohistochemistry have been identified in D.

**Figure 2:**
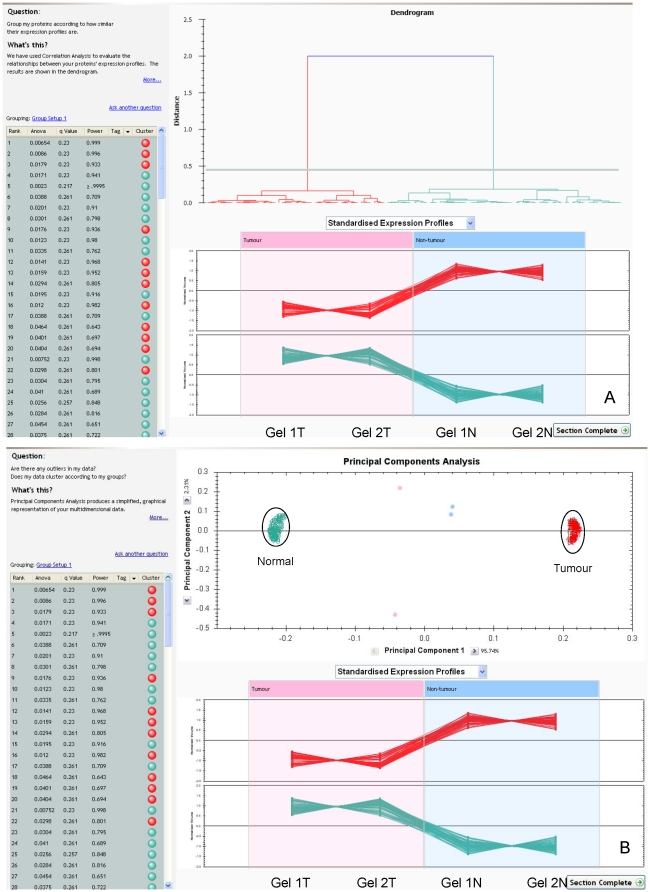
Hierarchical cluster and principal components analyses of 2D gels. Representative hierarchical cluster analysis (A) and principal components analysis (B) of normal and tumour gels. Both statistical methods show that the protein expression profiles determined by 2D gel electrophoresis and gel image analysis are distinct in tumour samples compared with normal samples. The lower panel in each figure shows the standardised expression profiles. The figures presented in figure 2 are “screen captures” of the output of analysis by the Progenesis SameSpots software. Both figure 2A and figure 2B represents the results of the same experiment of one case (i.e. one pair of normal gels N1 and N2 and one pair of tumour gels T1 and T2). The lower panel in each part of the figure shows the standardised expression profile and represents proteins of distinct expression plotted vertically with lines ”connecting” the corresponding proteins in each gel. The coloured spots represent interactive spots placed by the software for the user to access each data set and are positioned arbitrarily by the software on the screen.

In total more than 1200 individual protein spots were resolved following separation by 2D gel electrophoresis and image analysis in normal colonic mucosa and colon tumours (Figure 1). Hierarchical cluster analysis and principle components analysis showed the separation of the proteins into two distinct groups- normal and tumour (Figure 2). The study included both proximal and distal colon tumours and neither cluster nor principle components analysis showed that there was any difference in protein expression profiles between tumour and normal mucosa in these anatomical locations. Proteins showing greater than and equal to 1.5 fold increased expression in tumour samples are summarised in supporting information Table S2. The identity of these proteins was mostly confirmed by liquid chromatography–tandem mass spectrometry. For each protein multiple peptides with a high statistical probability (p<0.05) of matches to the relevant protein were analysed to confirm identity. Details of mass spectrometric identification of proteins are shown in supporting information Table S3.

### Immunohistochemistry

Fifteen proteins were selected for immunohistochemical validation. The criteria for the selection of the proteins included the degree of overexpression in colorectal cancer, exclusion of known structural e.g. actin and serum proteins e.g.haemoglobin and the availability of suitable validated antibodies which were effective on formalin fixed wax embedded tissue.

**Figure 3:**
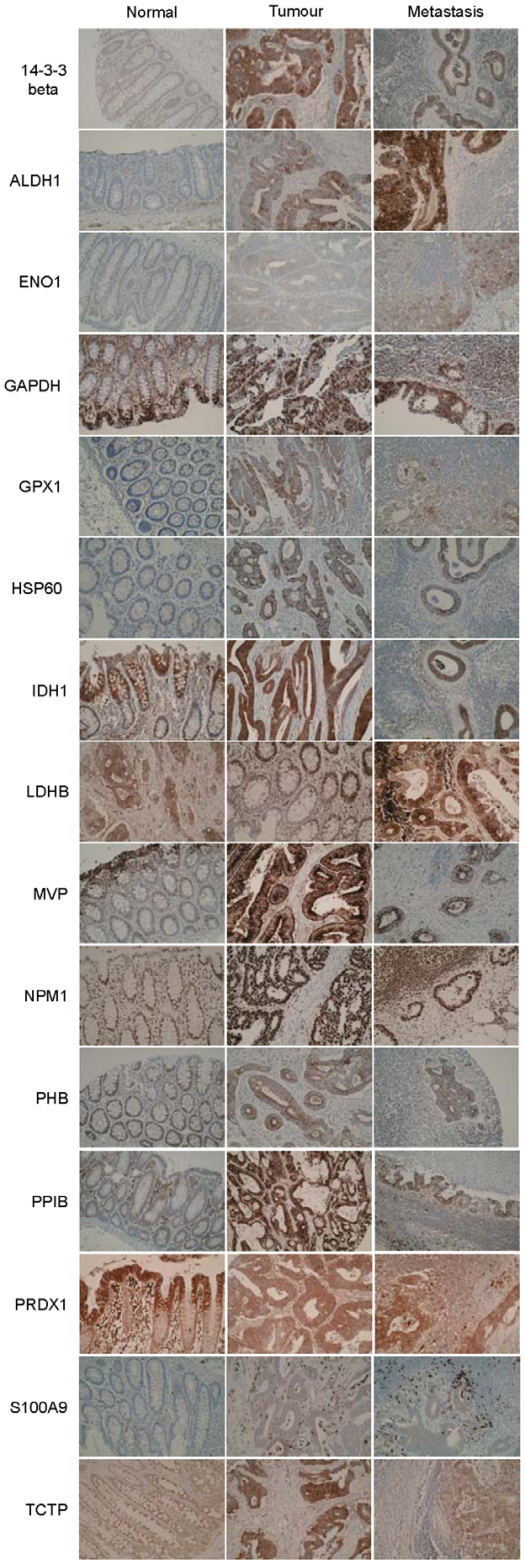
Immunohistochemistry photomicrographs. Photomicrographs of the immunohistochemical localisation of individual proteins in normal colon, primary colorectal cancer and lymph node metastasis.

**Figure 4:**
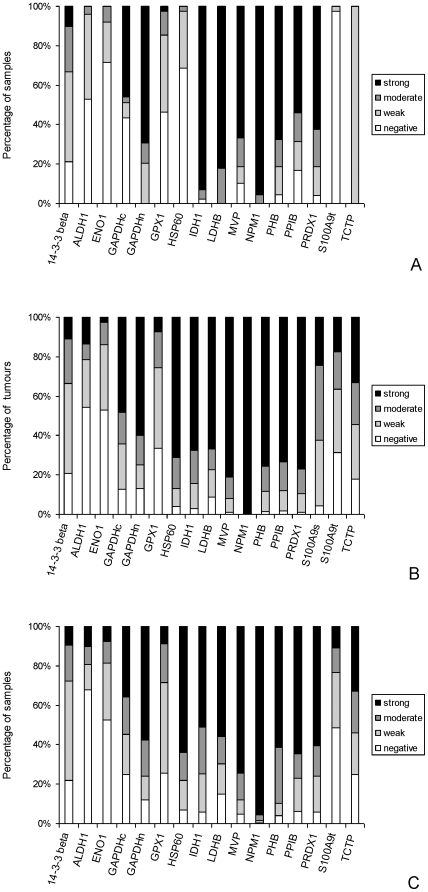
Frequency expression of individual proteins in normal colon, colorectal cancer and metastatic colorectal cancer. Frequency of expression as evaluated immunohistochemically of individual proteins in A. normal colon, B, primary colorectal cancer and C. lymph node metastasis.

All the proteins showed tumour cell staining and except nucleophosmin (NPM1) showed cytoplasmic staining (Figure 3). NPM1 showed exclusively nuclear staining while glyceraldehyde 3 phosphate dehydrogenase (GAPDH) showed both nuclear and cytoplasmic staining and these two sub-cellular localisations have been assessed separately for this protein. S100A9 showed both variable tumour cell staining (S100A9t) and variable stromal cell staining (S100A9s) and these two cellular localisations of this protein have been evaluated separately. The proteins that most frequently showed strong tumour cell immunoreactivity in primary colorectal cancer were NPM1 (99.6%), major vault protein (MVP, 81.1%) and prohibitin (PHB, 75.6%) while in lymph node metastasis those proteins which showed the most frequent strong tumour cell immunoreactivity were NPM1 (95.8%), MVP (74.5%) and heat shock protein 60 (HSP60, 63.9%) (Figure 4). In normal colon the proteins that showed the highest frequency of strong epithelial cell immunoreactivity were NPM1 (99.6%), isocitrate dehydrogenase 1 (IDH1, 93%) and lactate dehydrogenase B (LDHB, 82.1%) (Figure 4).

**Figure 5:**
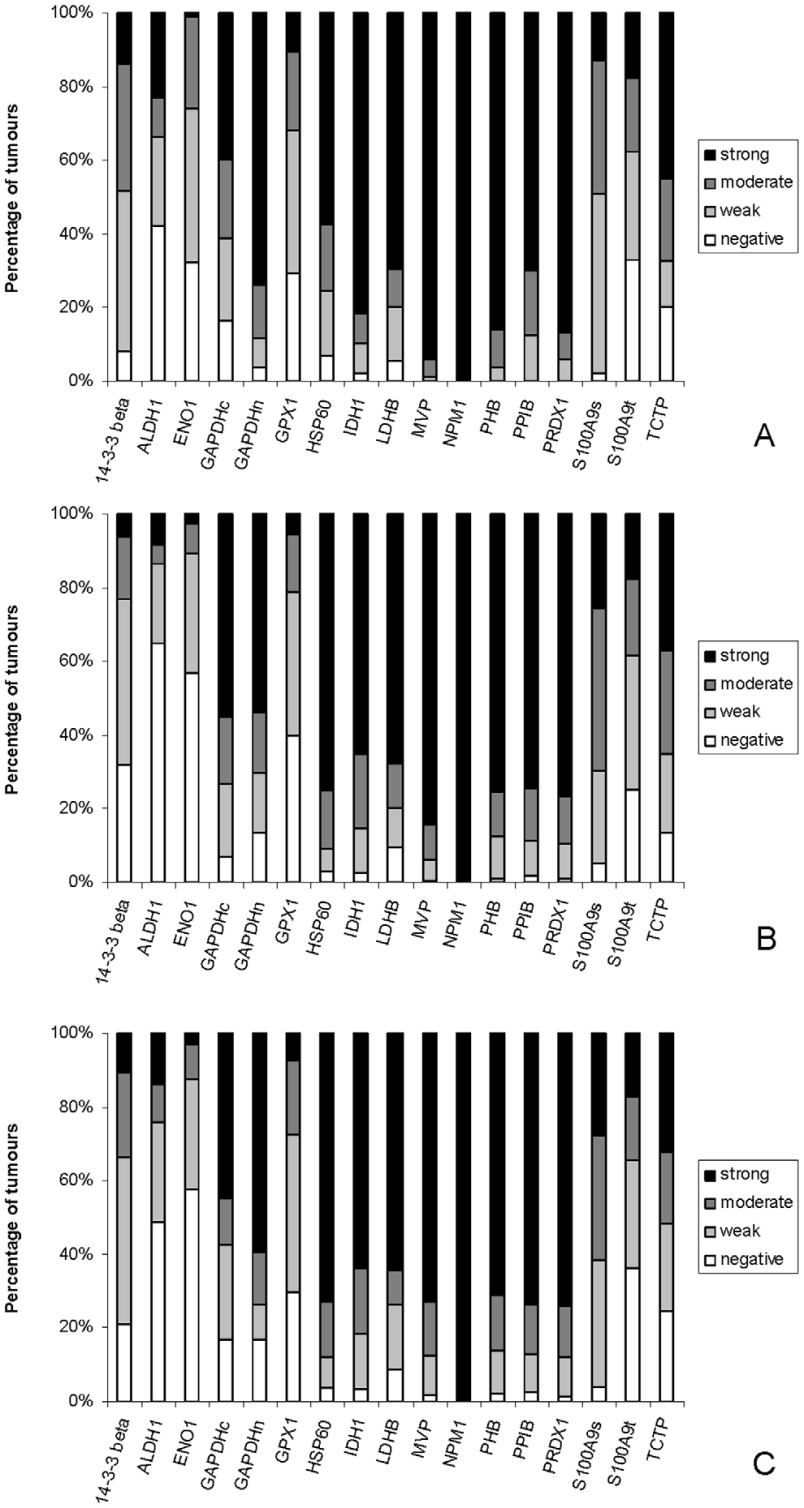
Frequency expression of individual proteins in specific Dukes stages of colorectal cancer. Frequency of expression of individual proteins as evaluated immunohistochemically in A. Dukes A colorectal cancer, B. Dukes B colorectal cancer and C. Dukes C colorectal cancer.

The proteins that showed the greatest degree of overexpression in primary colorectal cancer compared with normal colonic mucosa were HSP60 (p<0.001), S100A9 (p<0.001) and translatinally controlled tumour protein (TCTP, p<0.001, Table 3), while for Dukes C cancers no proteins showed increased immunoreactivity in the lymph node metastasis compared with the corresponding primary colorectal cancers and the proteins that showed the greatest decrease in expression in lymph node metastasis were PHB (p = 0.002), peroxiredoxin (PRDX1, p = 0.003) and HSP60 (p = 0.005, Table 3). The relationship of protein expression with individual Dukes stages is shown in Table 4 and Figure 5.

**Table 3:** Comparison of protein expression in normal colonic mucosa, primary colorectal cancer and lymph node metastasis.

Protein	Immunoreactivity (p value, normal *v* primary tumour)	Change in expression in tumour	Immunoreactivity (p value, primary Dukes C tumour *v* lymph node metastasis)	Change in expression in lymph node
14-3-3β	0.243	-	0.003	↓
ALDH1	0.328	-	0.005	↓
ENO1	0.015	↑	0.047	↓
GAPDHc	0.043	↑	0.074	-
GAPDHn	0.168	-	0.405	-
GPX1	0.053	↑	0.277	-
HSP 60	<0.001	↑	0.005	↓
IDH1	0.001	↓	0.02	↓
LDHB	0.015	↓	0.021	↓
MVP	0.009	↑	0.877	-
NPM1	0.003	↑	0.02	↓
PHB	0.056	-	0.002	↓
PPIB	0.001	↑	0.43	-
PRDX1	0.021	↑	0.003	↓
S100A9	<0.001	↑	0.009	↓
TCTP	<0.001	↑	0.74	-

Evaluation of normal colonic epithelium versus primary tumour samples for immunoreactivity (Mann-Whitney U test, ↑ = increased in tumour, ↓ = decreased in tumour, - = no change between tumour and normal) and evaluation of primary Dukes C colorectal tumour samples and their corresponding metastasis samples for immunoreactivity (Wilcoxon signed rank sum test, ↑ = increased in lymph node metastasis, ↓ = decreased in lymph node metastasis, - = no change between primary and metastatic tumour).

**Table 4:** The relationship of protein expression in Dukes A, Dukes B and Dukes C colorectal cancers (Mann-Whitney U test).

Protein	Dukes A v Dukes B (p value)	Dukes B v Dukes C (p value)
14-3-3β	<0.001	<0.001
ALDH1	<0.001	0.001
ENO1	0.001	0.960
GAPDHc	0.009	0.002
GAPDHn	0.001	0.443
GPX1	0.03	0.028
HSP 60	0.001	0.531
IDH1	<0.001	0.583
LDHB	0.714	0.413
MVP	0.02	0.004
NPM1	0.506	0.943
PHB	0.03	0.360
PPIB	0.517	0.795
PRDX1	0.053	0.521
S100A9s	<0.001	0.452
S100A9t	0.474	0.094
TCTP	0.562	0.014

Comparisons of the expression of individual proteins and clinico-pathological parameters are detailed in Table 5. Several of the proteins showed a highly significant association with microsatellite instability status including 14-3-3β (χ^2^ = 22.441, p<0.001), HSP60 (χ^2^ = 27.663, p<0.001) and IDH1 (χ^2^ = 47.733, p<0.001).

**Table 5:** Relationship of individual proteins with pathological parameters.

Pathological parameter														
	Tumour site		Tumour differentiation		EMVI		MSI status		T stage		N stage		Dukes stage	
Protein	χ^2^	p value	χ^2^	p value	χ^2^	p value	χ^2^	p value	χ^2^	p value	χ^2^	p value	χ^2^	p value
14-3-3β	8.306	0.217	2.887	0.409	4.806	0.187	**22.441**	**<0.001**	**18.234**	**0.033**	10.146	0.119	**35.370**	**<0.001**
ALDH1	12.495	0.052	5.318	0.15	0.509	0.917	7.090	0.069	**21.108**	**0.012**	8.858	0.182	**21.859**	**0.001**
ENO1	**14.941**	**0.021**	0.021	0.999	0.966	0.809	7.171.	0.067	**23.055**	**0.006**	8.895	0.18	**26.217**	**<0.001**
GAPDHc	5.225	0.515	5.817	0.121	3.238	0.356	2.969	0.396	12.92	0.166	11.138	0.084	**17.321**	**0.008**
GAPDHn	**20.89**	**0.002**	**13.442**	**0.004**	3.298	0.348	**12.516**	**0.006**	**17.227**	**0.045**	7.721	0.259	**17.678**	**0.007**
GPX1	4.841	0.564	1.018	0.797	6.848	0.077	4.185	0.282	6.027	0.737	5.198	0.519	7.733	0.258
HSP 60	12.318	0.055	3.965	0.265	4.826	0.185	**27.663**	**<0.001**	14.326	0.111	10.191	0.117	**14.548**	**0.024**
IDH1	7.912	0.245	**12.244**	**0.007**	0.464	0.927	**47.733**	**<0.001**	14.483	0.106	5.444	0.488	11.467	0.075
LDHB	6.046	0.418	0.015	0.999	7.529	0.057	**9.597**	**0.022**	10.23	0.332	5.679	0.46	5.53	0.478
MVP	7.86	0.249	**27.276**	**<0.001**	0.935	0.817	**21.393**	**<0.001**	12.053	0.21	**23.198**	**0.001**	**21.669**	**0.001**
NPM1	1.384	0.501	0.155	0.694	0.907	0.341	2.134	0.144	1.047	0.79	1.953	0.377	0.427	0.808
PHB	**15.693**	**0.016**	6.414	0.093	4.562	0.207	**21.392**	**<0.001**	**19.301**	**0.023**	5.072	0.535	9.468	0.149
PPIB	8.814	0.184	3.615	0.306	1.439	0.696	4.628	0.201	10.672	0.299	1.789	0.938	3.062	0.801
PRDX1	6.35	0.385	**8.636**	**0.035**	4.572	0.206	**9.808**	**0.020**	8.683	0.467	6.043	0.418	6.141	0.408
S100A9s	7.703	0.261	**9.12**	**0.028**	5.33	0.149	5.601	0.133	16.4	0.059	5.691	0.459	**19.979**	**0.003**
S100A9t	2.91	0.82	3.869	0.276	2.834	0.418	4.224	0.238	13.741	0.132	7.133	0.309	6.566	0.363
TCTP	**14.924**	**0.021**	5.115	0.164	2.569	0.463	**14.457**	**0.002**	5.86	0.754	**12.866**	**0.045**	**16.723**	**0.01**

Significant values are highlighted in bold.

### Survival analysis

#### Analysis of individual markers

**Figure 6:**
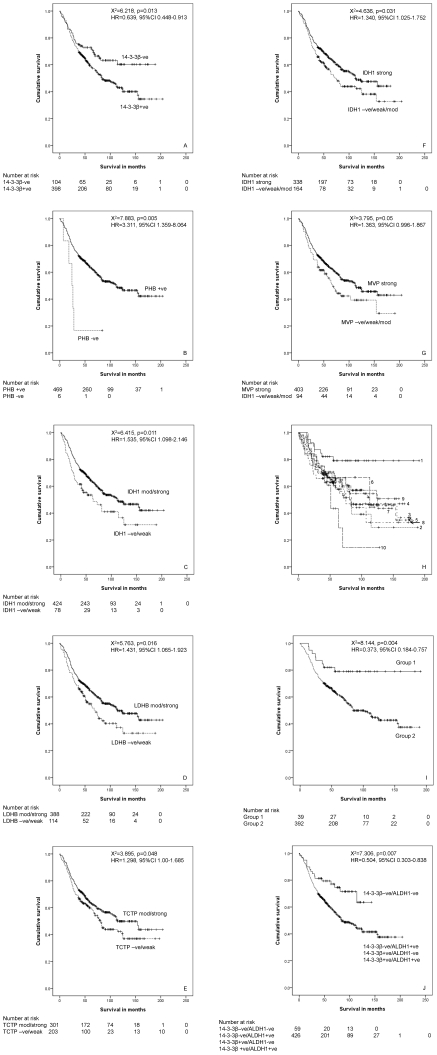
Survival curves of marker proteins. The relationship of individual proteins evaluated by immunohistochemistry with survival with different cut-off points. A. 14-3-3β (positive v negative immunoreactivity), B. PHB (positive v negative immunoreactivity), C. IDH1 (negative/weak immunoreactivity v moderate/strong immunoreactivity), D. LDHB (negative/weak immunoreactivity v moderate/strong immunoreactivity), E. TCTP (negative/weak immunoreactivity v moderate/strong immunoreactivity), F. IDH1 (negative/weak/moderate immunoreactivity v strong immunoreactivity), G. MVP (negative/weak/moderate immunoreactivity v strong immunoreactivity), H. survival in each of 10 clusters identified by hierarchical cluster analysis (each cluster is numerically identified and corresponds to the clusters that are identified in the cluster analysis panel of Figure 7), I. survival in 2 clusters- cluster 1 and clusters 2–10 combined and J. two protein signature of 14-3-3β and ALDH1 showing that double negative tumours have a significantly better outcome.

The relationship of the expression of individual proteins and survival was investigated using different cut-off points (negative v positive, negative/weak positive v moderate/strong and negative/weak/moderate v strong) and is summarised in Table 6 and Figure 6. 14-3-3β was identified as showing prognostic significance (χ^2^ = 6.218, p = 0.013, hazard ratio (HR) = 0.639, 95% confidence interval (CI) 0.448–0.913) when negative tumours were compared with tumours showing any degree of 14-3-3β immunoreactivity (Figure 6A). Tumours showing an absence of 14-3-3β immunoreactivity were associated with a better prognosis. For patients with 14-3-3β negative tumours (n = 104, number of deaths = 36) the mean survival was 129 months (95%CI 113–145 months) and for patients with positive tumours (n = 398; number of deaths = 194) the mean survival was 107 months (95% CI 98–117 months). This was prognostically significant (p = 0.03, HR = 0.588, 95%CI 0.361–0.958) in a multivariate model containing all variables (Dukes stage, EMVI, tumour site, patient age, patient sex, expression of individual proteins). The other significant variables were Dukes stage (p<0.001, HR = 0.491, 95%CI 0.357–0.714), age (p<0.001, HR = 0.470, 95%CI 0.343–0.645) and extramural vascular invasion (EMVI, p<0.001, HR = 0.467, 95%CI 0.338–0.644). Although PHB expression (positive v negative PHB immunoreactivity) was also noted to have a highly significant association with survival (χ^2^ = 7.883, p = 0.005, HR = 3.311, 95% CI 1.359–8.064) only 6 patients were in the PHB negative group (Figure 6B). Other protein which showed a significant relationship with survival using different cut off points were IDH1, LDHB, TCTP and MVP (Table 6 and Figure 6C–6G).

**Table 6:** The relationship of individual protein expression with survival (log rank test) using different cut-off points for the immunohistochemical data.

Cut-off point						
	Negative v weak/moderate/strong		Negative and weak v moderate and strong		Negative, weak and moderate *v* strong	
Protein	χ^2^	p value	χ^2^	p value	χ^2^	p value
14-3-3β	6.218	**0.013**	0.069	0.793	0.005	0.942
ALDH1	1.478	0.224	0.367	0.545	0.165	0.685
ENO1	1.758	0.185	2.323	0.127	0.47	0.493
GAPDHc	0.858	0.354	0.002	0.965	0.105	0.746
GAPDHn	1.653	0.198	0.10	0.922	0.490	0.484
GPX1	0.021	0.886	0.689	0.406	0.167	0.683
HSP 60	0.315	0.575	1.303	0.254	2.1	0.147
IDH1	0.094	0.759	6.415	**0.011**	4.636	**0.031**
LDHB	0.403	0.525	5.763	**0.016**	2.979	0.084
MVP	0.523	0.470	0.120	0.729	3.795	**0.051**
NPM1	-	-	-	-	0.009	0.923
PHB	7.883	**0.005**	0.237	0.627	0.249	0.617
PPIB	0.386	0.535	1.563	0.211	0.969	0.325
PRDX1	1.669	0.196	0.813	0.367	1.207	0.272
S100A9s	0.016	0.9	0.051	0.821	1.021	0.312
S100A9t	0.027	0.869	0.263	0.608	1.013	0.314
TCTP	0.341	0.559	3.895	**0.048**	3.776	0.052

Significant values are highlighted in bold.

#### Hierarchical cluster analysis and identification of prognostic protein signature

**Figure 7:**
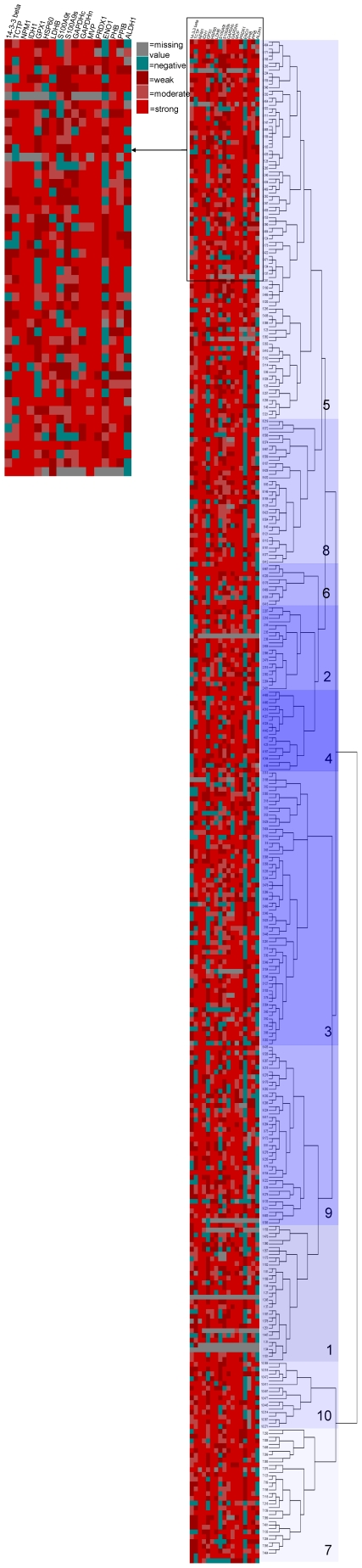
Hierarchical cluster analysis of immunohistochemical marker proteins. Graphical representation of the immunohistochemistry marker data is shown in the middle panel. The right hand panel shows the results of the hierarchical cluster analysis presented as a dendrogram with 10 individual clusters identified. The left hand panel shows an expanded segment of the graphical representation. Proteins are represented in columns and cases in rows.

Hierarchical cluster analysis was also used as an exploratory statistical tool to examine the overall relationship of marker expression with outcome and based on this identify a protein signature associated with prognosis. A range of cluster solutions (number of clusters) was investigated to determine the optimum number of clusters that produced groups with different outcomes. Clustering the data into ten clusters was identified as the optimum number of clusters for analysis in relation to the most prognostically significant groups (supporting information Table S4, Figure 6H and Figure 7). These 10 clusters were then combined into two prognostic groups; a good prognosis group (cluster 1) and a poor prognosis group (cluster groups 2–10) (Figure 6I). The good prognosis group (mean survival = 157 months 95% CI 135–177 months, n = 39, number of deaths = 8) had a significantly better survival (χ^2^ = 8.144, p = 0.004, HR = 0.373, 95% CI 0.179–0.757) than the poor prognosis group (mean survival = 106 months, 95%CI 1-2-119 months, n = 392, number of deaths = 183).

Further analysis of the data based on the distribution of proteins in these cluster groups identified a two protein signature of 14-3-3β and aldehyde dehydrogenase 1 (ALDH1) that showed greater prognostic significance (χ^2^ = 7.306, p = 0.007, HR = 0.504, 95%CI 0.303–0.838) than 14-3-3β alone (Figure 6J). Those tumours that were both 14-3-3β and ALDH1 negative had a better prognosis than tumours showing either 14-3-3β or ALDH1 positivity. For patients with 14-3-3 β/ALDH1 negative tumours (n = 59, number of deaths = 16) the mean survival (estimate) was 109 months (95%CI 95–123 months) and for patients with either or both 14-3-3 β and ALDH1 positive tumours (n = 426, number of deaths = 206) the mean survival was 110 months (95% CI 101–119 months). This was also prognostically significant (p = 0.01, HR = 0.416, 95%CI 0.208–0.829) in a regression model containing all variables (Dukes stage, EMVI, tumour site, tumour differentiation, patient age, patient sex, 14-3-3 β/ALDH1 expression). The other significant variables were Dukes stage (p<0.001, HR = 0.492, 95%CI = 0.338–0.715), age (p<0.001, HR = 0.463, 95% CI 0.337–0.636) and EMVI (p<0.001, HR = 0.484, 95%CI 0.351–0.668).

## Discussion

This study has performed a comprehensive proteomic analysis and immunohistochemical validation of protein expression in a large well characterised series of colorectal cancers (n = 515). The overexpression of individual proteins in colorectal cancer has been established and a two protein signature associated with prognosis identified.

There have been a number of proteomic studies performed on colorectal cancer. A range of proteomics technology have been utilised although the predominant technologies have been 2D gel electrophoresis combined with mass spectrometry which are both robust and well established technologies [32]–[42]. In most of those proteomics studies usually only a small number (often less than 10) of tissue samples have been included and some of the tissue samples included in these studies have little or no clinico-pathological information possibly as a consequence of the samples having been procured from a third party tissue or tumour bank. In the absence of the clinico-pathological information interpretation of the proteomic studies is more difficult. Similarly when a validation component has been incorporated into those studies these have often been limited by the number of samples included in this part of individual investigations [41].

Proteomics showed that the most significantly overexpressed in protein in colorectal cancer was the beta sub-unit of 14-3-3. The 14-3-3 proteins are phosphoserine/phosphothreonine binding proteins composed of seven subunits which can both homo- and heterodimerise [43]–[45]. These proteins are involved in the regulation of multiple cellular signalling pathways including cell cycle regulation, apoptosis, metabolism, transcription and protein trafficking many of them in a phosphorylation dependent manner. They are known to interact with pathways e.g. ras/raf and AKT/mTOR pathways involved in tumourigenesis [45]. Other proteins that were shown to be ovexpressed in CRC included the metabolic enzymes (enolase 1 (ENO1), GAPDH, IDH1 and LDHB) involved in pathways of glucose metabolism. Some of these proteins have previously been noted to have increased expression in CRC by proteomics [41] and highlights the increased/altered glucose metabolism occurring in tumours [46].

The selection of proteins to be validated by immunohistochemistry was based on the degree of overexpression identified by the proteomic studies with the exclusion of structural and serum proteins and the availability of well characterised antibodies already shown to be effective on formalin fixed wax embedded tissue. The presence of 14-3-3β in colorectal cancer samples was confirmed by immunohistochemistry with a cytoplasmic location in tumour cells although its overexpression was not substantiated by immunohistochemistry. However, the comparative evaluation of proteins is based on different technologies. 2D gel electrophoresis and images analysis identified and compares average spot volumes in a gel while immunohistochemistry identifies cellular/subcellular location of the protein combined with a semi-quantitative assessment of the intensity of immunoreactivity of the individual protein.

Two methods were used to explore the relationship of protein expression with clinico-pathological factors and outcome. Each marker was assessed independently as a discrete variable in univariate survival analysis while hierarchical cluster analysis was performed to explore the overall relationship of marker expression, clinicopathological factors and survival to provide a more detailed understanding of that relationship.

The relation of individual proteins with survival in univariate analysis was explored in the data set using different cut-off points to dichotomize the data. The most robust cut-off point would appear to be the division between absence and presence of immunoreactivity when considered on the likelihood of reproducibility. On that basis 14-3-3β was associated with prognosis with absent 14-3-3β being associated with a better prognosis. The use of other cut-off points highlight other potential markers (IDH1, LDHB, MVP, PHB and TCTP) however those cut-off points i.e. a division between weak and moderate staining or a division between moderate and strong staining are potentially much less robust in practice than a cut-off between negative and positive.

Hierarchical cluster analysis which has been widely applied to gene expression data sets but rarely immunohistochemical data [18], [19] identified multiple clusters and based on cluster membership the combination of two proteins were identified namely 14-3-3β and ALDH1 as prognostically significant. It is interesting to note that ALDH1 has been proposed as a stem cells marker and has recently been suggested to be a marker of colon cancer stem cell [47].

The colorectal cancer tissue microarray was also specifically designed to include lymph node metastasis from those primary tumours with lymph node metastasis. This is a particular strength of the design of this tumour microarray and allowed a direct comparison of the phenotype of primary tumours and their synchronous lymph node metastasis. This is important for example as treatment in the adjuvant setting is targeted at metastatic disease and it is an assumption that the phenotype of primary tumours necessarily reflects the phenotype of secondary tumours [48], [49]. Expression in the metastasis is likely to be influence by the microenvironmental setting in which the metastasis develop [47]. Most of the proteins examined showed decreased expression in the metastasis compared with their corresponding primary tumours indicating that further deregulation of protein expression is occurring in the lymph node metastasis. Most notably 14-3-3β, ALDH1 and PHB showed significant decreases in expression in lymph node metastasis compared with primary tumours providing evidence for further dysregulation of protein expression in metastasis [47].

In summary this study has performed a comprehensive proteomics analysis of colorectal cancer and identified proteins that are overexpressed in colorectal cancer. Validation has been performed using immunohistochemistry and a two-protein signature associated with prognosis identified.

## Supporting Information

Table S1 Clinico-pathological characteristics of the patients included in the colorectal cancer tissue microarray.
(PDF)

Table S2 List of proteins which showed significantly increased expression in colon cancer (≥1.5 fold).
(PDF)

Table S3 Details of mass spectrometric analysis of individual proteins.
(PDF)

Table S4 Relationship of survival of individual clusters identified by cluster analysis.
(PDF)
