# Supplementary material for: The Proteomics of Colorectal Cancer: Identification of a Protein Signature Associated with Prognosis
Source: PLoS One. 2011 Nov 18;6(11):e27718. doi: 10.1371/journal.pone.0027718 (PMC3220687; doi:10.1371/journal.pone.0027718)
Supplement: Table S1 — Clinico-pathological characteristics of the patients included in the colorectal cancer tissue microarray. (PDF) [file pone.0027718.s001.pdf]

Table S1. Clinico-pathological characteristics of the patients included in the colorectal cancer tissue microarray

		Percent (number)	Mean survival (months, 95% CI),	Relationship with survival, hazard ratio and 95% CI
Sex				
	Male	51% (263)	112 (100-123)	
	Female	49% (252)	110 (100-121)	
				χ^2 =0.187; p=0.665, HR=0.972 (0.856-1.105)
Age (mean:range)		(69:33-94)		
	< 70	48% (245)	135 (124-147)	
	≥ 70	52% (270)	87 (78-96)	
				χ^2 =20.167; p<0.001, HR=0.551 (0.423-0.718)
Tumour site*				
	Colon	75% (389)	111 (101-120)	
	Proximal	38% (198)	102 (89-116)	
	Distal	37% (191)	117 (105-130)	
	Rectum	25% (126)	117 (102-133)	
				colon v rectum χ^2 = 0.066; p=0.798, HR=1.019 (0.881-1.179)<br>proximal v distal χ^2 = 5.858; p=0.016, HR=1.441 (1.069-1.943)<br>proximal v rectum χ^2 =1.638; p=0.201, HR=1.231 (0.894-1.697)<br>distal v rectum χ^2 =0.649; p=0.421, HR=0.872 (0.624-1.219)
Tumour differentiation				
	Well/moderate	92% (476)	116 (107-124)	
	Poor	8% (39)	84 (61-109)	
				χ^2 =3.659; p=0.056, HR=1.231 (0.993-1.527)
Tumour stage				
	T1	4.7.% (24)	128 (101-154)	
	T2	16.3% (84)	168 (152-183)	
	T3	65.6% (338)	103 (95-103)	
	T4	13.4% (69)	52 (39-64)	
				T1vT2, χ^2 =2.175; p=0.140, HR=1.868 (0.802-4.348)<br>T2vT3, χ^2 =25.477; p<0.001, HR=0.298 (0.180-0.491)<br>T3vT4, χ^2 =25.048; p<0.001, HR=0.453 (0.329-0.624)

Nodal stage				
	N0	56.5% (291)	148 (139-159)	
	N1	27.4% (141)	76 (63-89)	
	N2	16.1% (83)	51 (39-63)	
				N0vN1, χ^2 =75.180; p<0.001, HR=0.286 (0.211-0.386)<br>N1vN2, χ^2 =7.294; p=0.007, HR=0.649 (0.472-0.893)
EMVI				
	Present	21.9% (113)	54 (44-65)	
	Absent	78.1% (402)	130 (121-140)	
				χ^2 =87.078; p<0.001, HR=0.305 (0.234-0.398)
Microsatellite instability (MSI) status				
	Intact	82.9% (498)	116 (107-126)	
	Defective	13.8% (71)	96 (77-115)	
	Not assessable	3.3% (17)		
				χ^2 =1.526; p=0.217, HR=1.118 (0.936-1.336)
Screen Detected				
	Yes	6.8% (35)	116 (107-124)	
	No	93.2% (480)	109 (101-118)	
				χ^2 =15.369; p<0.001, HR=2.648 (1.498-4.680)
Stage				
	Dukes A, (Stage 1; T1/T2, N0)	17.5% (90)	165 (151-181))	
	Dukes B, (Stage 2; T3/T4, N0)	39.0% (201)	124 (114-134)	
	Dukes C, (Stage 3; any T stage and N1 or N2)	43.5% (224)	68 (58-78)	
				Dukes A v Dukes B, χ^2 =6.232; p=0.013, HR=0.524 (0.312-0.878)<br>Dukes B v Dukes C, χ^2 =70.578; p<0.001, HR=0.303 (0.225-0.407)

*Proximal colon tumours arose proximal to the splenic flexure (i.e. caecum ,ascending colon, transverse colon) while distal colon tumours arose distal to this point (i.e. splenic flexure, descending colon and sigmoid colon).
