# Supplementary material for: The Proteomics of Colorectal Cancer: Identification of a Protein Signature Associated with Prognosis
Source: PLoS One. 2011 Nov 18;6(11):e27718. doi: 10.1371/journal.pone.0027718 (PMC3220687; doi:10.1371/journal.pone.0027718)
Supplement: Table S2 — List of proteins which showed significantly increased expression in colon cancer (≥1.5 fold). (PDF) [file pone.0027718.s002.pdf]

Table S2. List of proteins which showed significantly increased expression in colon cancer (≥1.5 fold).

Spot id	Protein	Fold increase	p-value (anova)	Average normalised volumes	
				Normal	Tumour
ID: 0005	**14-3-3β**	2.3	7.985e-007	1324831.940	3109145.873
ID: 0006	Fibrinogen fragment D, chain B	2.3	1.206e-005	2836930.597	6584496.116
ID: 0879	**Enolase 1**	2.3	8.906e-010	907223.142	2085635.695
ID: 0392	B-actin	2.2	2.505e-009	4181454.163	9283387.640
ID: 0752	**Isocitrate dehydrogenase 1**	2.2	0.039	561275.204	1244265.117
ID: 0553	**Nucleophosmin**	2.1	4.067e-011	3580652.430	7658032.639
ID: 1016	Glia maturation factor γ	2.0	3.408e-005	2578532.891	5208661.695
ID: 1080	**Glutathione peroxidase**	2	0.0003571	394012.027	786887.734
ID: 0033	Isocitrate dehydrogenase (NADP+)	1.9	0.0002757	3209117.169	6245612.597
ID: 0560	Dynactin 3 isoform 1	1.9	0.0000001361	2314461.486	4456363.372
ID: 1078	**Prohibitin (PHB)**	1.9	0.00006552	6904774	13148679.672
ID: 0511	Serum albumin	1.9	0.00001834	2145360.256	4020882.581
ID: 0412	14-3-3 zeta	1.8	3.968e-009	2675769.019	4928813.139
ID: 0913	Eosinophil lysophospholipase	1.8	0.00001233	2239373.575	4093490.947
ID: 0939	**Peptidylprolyl isomerase B (PPIB)**	1.8	0.00004559	19544099.009	34450992.284
ID: 0848	**Peroxiredoxin 1 (PRDX1)**	1.7	0.00000003612	5218358.001	9129146.788
ID: 1116	**Major vault protein (MVP)**	1.7	0.00005321	1470725.174	2570269.447
ID: 0535	14-3-3 gamma	1.7	0.0001325	563724.535	981311.416
ID: 1213	Haptoglobin precursor	1.7	0.0000174	6439426.779	11194985.25
ID: 0187	COP9 complex subunit 4	1.7	0.0002206	1089758.828	1869157.705
ID: 1010	Haemoglobin, chain B	1.7	2.485e-005	17065055.168	28861645.381
ID: 1058	**Lactate dehydrogenase B (LDHB)**	1.7	0.0000002496	672741.116	1119332.379
ID: 0956	S100 calcium-binding protein A8	1.7	0.003	7720491.797	12830081.36

Spot id	Protein	Fold increase	p-value (anova)	Average normalised volumes	
				Normal	Tumour
ID: 0186	zinc ribbon domain containing 1	1.6	0.00005876	368009.008	603932.211
ID: 0820	**S100 calcium binding protein A9 (S100A9)**	1.6	0.0006801	1480786.115	2396949.799
ID: 0821	Immunoglobulin	1.6	5.577e-009	1572964.363	2544716.182
ID: 0193	**Glyceraldehyde-3-phosphate dehydrogenase (GAPDH)**	1.6	2.820e-005	6407144.166	10354673.492
ID: 1046	Cofilin 1 (non-muscle)	1.6	0.001	11923877.211	19247273.763
ID: 0554	**Translationally controlled tumour protein (TCTP)**	1.6	1.122e-009	1841008.184	2955603.433
ID: 0702	Myotrophin	1.6	0.00007419	1319337.224	2080318.329
ID: 0541	**Heat shock protein 60 (HSP60)**	1.6	0.000001944	1784776.361	2812575.396
ID: 0690	Proteasome	1.6	3.112e-007	2520088.604	3935590.753
ID: 1132	Nm23 protein (nucleoside diphosphate kinase A)	1.5	0.00000009724	1762743.158	2725563.671
ID: 0306	S-adenosylhomocysteine hydrolase	1.5	0.00009597	8768209.754	13371451.083
ID: 0759	Tropomyosin 1 alpha chain isoform 5	1.5	0.0000079	4285922.056	6528206.612
ID: 0516	Galectin-4	1.5	0.018	11435503.643	17392922.066
ID: 0631	**Aldehyde dehydrogenase 1 (ALDH1)**	1.5	9.887e-007	2915660.691	4413957.528
ID: 1055	Lysosomal pepstatin insensitive protease	1.5	0.0001618	13809241.088	20545430.845
ID: 0458	Transgelin 2	1.5	0.01	2178940.99	3215570.618
ID: 0279	Ornithine aminotransferase	1.5	0.008	1254863.974	1838628.107
ID: 1209	Tropomyosin 4 isoform 1	1.5	0.0003236	1752843.046	2541820.128

The spot id corresponds to the numbering shown in figure 1 of the annotated gels. Proteins showing a significant increase in tumours are shown and listed according to fold increase in tumour samples. Average normalised spot volumes in tumour and normal are also shown. The proteins highlighted in bold were validated by immunohistochemistry.
