# Supplementary material for: The Proteomics of Colorectal Cancer: Identification of a Protein Signature Associated with Prognosis
Source: PLoS One. 2011 Nov 18;6(11):e27718. doi: 10.1371/journal.pone.0027718 (PMC3220687; doi:10.1371/journal.pone.0027718)
Supplement: Table S3 — Details of mass spectrometric analysis of individual proteins. (PDF) [file pone.0027718.s003.pdf]

Supporting information table S1. Details of proteins and individual peptides identified by mass spectrometry.

Protein	Accession[1]	Mr (calc)[2]	pI (calc)[2]	Protein score[3]	Coverage[4]	Peptide sequence[5]	m/z[6]	Charge	Ion score[7]	Expect[8]
14-3-3 beta	gi|4507949	28179	4.76	1217	84%	LAEQAER	408.786	2+	58	0.0025
						VISSIEQK	452.330	2+	45	0.041
						NLLSVAYK	454.297	2+	51	0.0099
						YDDMAAAMK	508.298	2+	65	0.00042
						YLSEVASGDNK	591.914	2+	54	0.004
						DSTLIMQLLR	595.501	2+	64	0.00042
						YLIPNATQPESK	681.033	2+	65	0.00031
						AVTEQGHELSNEER	800.054	2+	83	4.2E-06
						IEAELQDICNDVLELLDK	1065.799	2+	87	2.0E-06
						TAFDEAIAELDTLNEESYK	1080.196	2+	125	3.3E-10
						QTTVSNSQQAYQEAFEISK	1080.297	2+	91	8.8E-07
						LGLALNFSVFYYEILNSPEK	1159.352	2+	117	1.1E-09
						DNLTLWTSENQGDEGDAGEGEN	1176.194	2+	106	1.3E-08
						EKIEAELQDICNDVLELLDK	1194.341	2+	115	1.7E-09
Fibrinogen fragment D, chain B	gi|2781208	38081	5.84	1859	85%	QDGSVDFGR	490.762	2+	60	0.0011
						EDGGGWWYNR	620.341	2+	47	0.021
						DNDGWLTSDPR	638.348	2+	63	0.0006
						AHYGGFTVQNEANK	768.455	2+	90	1.0E-06
						HGTDDGVVWMNWK	772.938	2+	91	7.5E-07
						LESDVSAQMEYCR	794.408	2+	97	1.8E-07
						TPCTVSCNIPVVSGK	809.965	2+	63	0.0005
						YYWGGQYTWDMAK	842.925	2+	89	1.1E-06
						MGPTELLIEMEDWK	854.515	2+	107	1.6E-08
						DNENVVNEYSSELEK	884.947	2+	85	2.9E-06
						NYCGLPGEYWLGNDK	893.509	2+	60	0.00095
						IQKLESDVSAQMEYCR	653.054	3+	66	0.00019
						MGPTELLIEMEDWKGDK	670.107	3+	55	0.0021
						HQLYIDETVNSNIPTNLR	1064.111	2+	96	2.5E-07
						VYCDMNTENGGWTVIQNR	1079.074	2+	101	7.7E-08
						GGETSEMYLIQPDSSVKPYR	753.093	3+	63	0.00029
						AHYGGFTVQNEANKYQISVNK	1184.669	2+	64	0.00026
						KGGETSEMYLIQPDSSVKPYR	801.096	3+	83	2.5E-06
						TPCTVSCNIPVVSGKECEEIIR	850.134	3+	77	1.3E-05

Protein	Accession[1]	Mr (calc)[2]	pI (calc)[2]	Protein score[3]	Coverage[4]	Peptide sequence[5]	m/z[6]	Charge	Ion score[7]	Expect[8]
Enolase 1	gi|4503571	47481	7.01	1351	61%	IGAEVYHNLK	381.914	3+	48	0.02
						LMIEMDGTENK	648.843	2+	62	0.00061
						YISPDQLADLYK	713.489	2+	66	0.00024
						FGANAILGVSLAVCK	760.593	2+	56	0.0024
						VVIGMDVAASEFFR	779.036	2+	117	1.7E-09
						IDKLMIEMDGTENK	834.970	2+	60	0.00091
						SKFGANAILGVSLAVCK	579.110	3+	70	8.2E-05
						AAVPSGASTGIYEALELR	903.145	2+	119	1.0E-09
						LAMQEFMILPVGAANFR	970.592	2+	97	1.4E-07
						DATNVGDEGGFAPNILENK	654.417	3+	51	0.0048
						FTASAGIQVVGDDLTVTNPK	1017.170	2+	73	3.0E-05
						DYPVVSIEDPFDQDDWGAWQK	1256.130	2+	74	2.0E-05
						SFIKDYPVVSIEDPFDQDDWGAWQK	995.876	3+	54	0.0016
						HIADLAGNSEVILPVPAFNVINGGSHAG	1004.661	3+	46	0.029
Beta actin	gi|4501885	42052	5.29	921	42%	AGFAGDDAPR	488.821	2+	60	0.0014
						DLTDYLMK	499.791	2+	50	0.0098
						GYSFTTTAER	566.861	2+	75	3.3E-05
						HQGVMVGMGQK	586.312	2+	85	3.2E-06
						DSYVGDEAQSK	599.875	2+	75	3.4E-05
						DSYVGDEAQSKR	677.907	2+	80	1.1E-05
						IWHHTFYNELR	758.465	2+	56	0.0025
						SYELPDGQVITIGNER	896.088	2+	97	3.7E-07
						VAPEEHPVLLTEAPLNPK	977.643	2+	63	0.0004
						LCYVALDFEQEMATAASSSSLEK	1283.661	2+	184	1.9E-16
Isocitrate dehydrogenase 1	gi|28178825	46915	6.53	1328	79%	NILGGTVFR	488.835	2+	48	0.024
						DATNDQVTK	496.276	2+	45	0.04
						HAYGDQYR	505.253	2+	44	0.04
						SEGGFIWACK	577.813	2+	67	0.00022
						LIDDMVAQAMK	617.898	2+	93	6.2E-07
						TVEAEAAHGTVTR	671.378	2+	86	2.5E-06
						VEITYTPSDGTQK	719.928	2+	57	0.0019
						FKDIFQEIYDK	723.407	2+	68	0.00014
						SDYLNTFEFMDK	755.410	2+	85	3.6E-06
						ISGGSVVEMQGDEMTR	848.451	2+	87	1.5E-06
						SIEDFAHSSFQMALSK	899.545	2+	64	0.00037
						GQETSTNPIASIFAWTR	940.077	2+	113	3.8E-09
						VTYLVHNFEEGGGVAMGMYNQDK	859.466	3+	64	0.00054
						ELAFFANALEEVSIETIEAGFMTK	892.923	3+	70	5.3E-05

Protein	Accession[1]	Mr (calc)[2]	pI (calc)[2]	Protein score[3]	Coverage[4]	Peptide sequence[5]	m/z[6]	Charge	Ion score[7]	Expect[8]
Nucleophosmin	gi|825671	31090	4.71	311	27%	VDNDENEHQLSLR	784.962	2+	87	2.0E-06
						DELHIVEAEAMNYEGSPIK	721.081	3+	73	3.4E-05
Glutathione peroxidase	gi|4902773	22181	8.41	188	45%	DFTQLNELQCR	712.434	2+	71	7.1E-05
Dynactin 3	gi|6005745	21220	5.37	80	15%	LAQIHIQQQDQCVEITEESK	799.813	3+	72	3.5E-05
Serum albumin	gi|62113341	71092	5.85	790	18%	FQNALLVR	480.795	2+	67	0.00025
						HPDYSVVLLLR	656.456	2+	70	9.7E-05
						RHPDYSVVLLLR	734.512	2+	66	0.00024
						VPQVSTPTLVEVSR	756.510	2+	82	6.4E-06
						CCAAADPHECYAK	776.875	2+	59	0.0012
						DVFLGMFLYEYAR	820.513	2+	93	3.9E-07
						KVPQVSTPTLVEVSR	820.539	2+	82	5.7E-06
						QNCELFEQLGEYK	829.492	2+	92	5.0E-07
						VFDEFKPLVEEPQNLIK	1023.120	2+	63	0.00032
						QNCELFEQLGEYKFQNALLVR	867.256	3+	49	0.017
14-3-3 zeta	gi|4507953	27899	4.73	325	32%	DSTLIMQLLR	603.381	2+	51	0.0082
						YLAEVAAGDDKK	640.394	2+	57	0.002
						SVTEQGAELSNEER	774.919	2+	92	6.0E-07
						GIVDQSQQAYQEAFEISK	681.054	3+	72	4.6E-05
Peroxiredoxin 1	gi|4505591	22324	8.27	452	46%	ADEGISFR	447.719	2+	50	0.013
						GLFIIDDK	460.778	2+	45	0.044
						DISLSDYK	470.734	2+	45	0.035
						TIAQDYGVLK	554.345	2+	54	0.0049
						ATAVMPDGQFK	590.793	2+	44	0.042
						QITVNDLPVGR	606.407	2+	53	0.0052
						QGGLGPMNIPLVSDPK	820.031	2+	60	0.0008
Major vault protein	gi|5851638	19313	5.21	760	88%	MVTVPPR	408.250	2+	46	0.036
						HYCTVANPVSR	652.454	2+	69	0.00015
						DAQGLVLFDVTGQVR	809.650	2+	97	1.8E-07
						KEVEVVEIIQATIIR	870.728	2+	125	2.4E-10
						LAQDPFPLYPGEVLEK	908.683	2+	73	4.0E-05
						VVAGDEWLFEGPGTYIPR	1003.747	2+	97	1.4E-07
						IPPYHYIHVLDQNSNVSR	718.224	3+	63	0.00034

Protein	Accession[1]	Mr (calc)[2]	pI (calc)[2]	Protein score[3]	Coverage[4]	Peptide sequence[5]	m/z[6]	Charge	Ion score[7]	Expect[8]
14-3-3 gamma	gi|9507245	28456	4.80	445	34%	LAEQAER	408.880	2+	52	0.01
						NLLSVAYK	454.274	2+	49	0.017
						NLLSVAYKNVVGAR	752.565	2+	81	6.7E-06
						NVTELNEPLSNEER	822.461	2+	99	1.1E-07
						ELEAVCQDVLSLLDNYLIK	745.585	2+	50	0.0076
Haptoglobin precursor	gi|306882	45860	6.24	414	21%	VGYVSGWGR	490.759	2+	64	0.00045
						VTSIQDWVQK	602.386	2+	78	2.0E-05
						SCAVAEYGVYVK	673.387	2+	81	8.3E-06
						YVMLPVADQDQCIR	862.449	2+	95	2.8E-07
						SPVGVQPILNEHTFCAGMSK	724.764	3+	55	0.002
Haemoglobin, chain B	gi|229752	15971	6.81	757	90%	SAVTALWGK	466.807	2+	47	0.027
						LHVDPENFR	563.799	2+	55	0.0033
						VVAGVANALAHK	575.392	2+	68	0.00016
						LLVVYPWTQR	637.951	2+	67	0.00023
						VNVDEVGGEALGR	657.913	2+	92	6.1E-07
						GTFATLSELHCDK	739.890	2+	65	0.00027
						VLGAFSDGLAHLDNLK	835.517	2+	87	1.6E-06
						LLGNVLVCVLAHHFGK	889.095	2+	54	0.0057
						KVLGAFSDGLAHLDNLK	600.037	3+	62	0.00064
						FFESFGDLSTPDAVMGNPK	1038.087	2+	95	1.9E-07
Lactate dehydrogenase B	gi|4557032	36900	5.71	1290	64%	LNLVQR	371.712	2+	44	0.039
						IVVVTAGVR	457.288	2+	76	3.0E-05
						GLTSVINQK	480.281	2+	54	0.0047
						LKDDEVAQLK	579.856	2+	61	0.00089
						VIGSGCNLDSAR	624.855	2+	84	4.1E-06
						MVVESAYEVIK	634.408	2+	89	1.1E-06
						IVADKDYSVTANSK	755.929	2+	91	8.7E-07
						SLADELALVDVLEDK	815.555	2+	101	8.2E-08
						LIAPVAEEEATVPNNK	847.993	2+	64	0.00037
						SLADELALVDVLEDKLK	936.195	2+	50	0.0081
						GEMMDLQHGSLFLQTPK	655.417	3+	56	0.002
						ITVVGVGQVGMACAISILGK	987.113	2+	104	4.1E-08
						GYTNWAIGLSVADLIESMLK	733.108	3+	60	0.00058
						LKGEMMDLQHGSLFLQTPK	735.785	3+	46	0.017
						GMYGIENEVFLSLPCILNAR	1156.634	2+	81	6.5E-06
						LKGYTNWAIGLSVADLIESMLK	808.268	3+	73	2.7E-05

Protein	Accession[1]	Mr (calc)[2]	pI (calc)[2]	Protein score[3]	Coverage[4]	Peptide sequence[5]	m/z[6]	Charge	Ion score[7]	Expect[8]
S100 calcium-binding protein A8	gi|21614544	10885	6.51	635	100%	GADVWFK	411.741	2+	47	0.023
						MLTELEK	440.212	2+	45	0.047
						GNFHAVYR	482.260	2+	48	0.022
						ALNSIIDVYHK	636.860	2+	62	0.00069
						LLETECPQYIR	710.909	2+	73	5.2E-05
						LLETECPQYIRK	775.440	2+	53	0.051
						KLLETECPQYIR	775.478	2+	71	6.8E-05
S100 calcium binding protein A9	gi|4506773	13291	5.71	388	57%	KDLQNFLK	503.260	2+	48	0.02
						LGHPDTLNQGEFK	728.381	2+	58	0.0014
						QLSFEEFIMLMAR	824.014	2+	100	9.5E-08
						NIETIINTFHQYSVK	904.032	2+	89	1.1E-06
Immunoglobulin kappa light chain	gi|21669345	28918	6.15	362	41%	DSTYSLSSTLTLSK	751.986	2+	65	0.00028
						FSGSGSGTDFTLTISR	816.974	2+	86	2.3E-06
						SGTASVVCLLNNFYPR	899.597	2+	77	2.1E-05
						TVAAPSVFIFPPSDEQLK	649.446	3+	47	1.7E-02
						VDNALQSGNSQESVTEQDSK	1068.534	2+	61	0.00053
Glyceraldehyde-3-phosphate dehydrogenase	gi|31645	36202	8.26	227	14%	VPTANVSVVDLTCR	765.979	2+	103	4.3E-08
						LISWYDNEFGYSNR	882.935	2+	77	1.8E-05
Cofilin 1 (non-muscle)	gi|5031635	18719	8.22	303	39%	YALYDATYETK	669.397	2+	56	0.0027
						EILVGDVGQTVDDPYATFVK	1083.597	2+	114	2.8E-09
						NIILEEGKEILVGDVGQTVDDPYATFVK	1021.677	3+	55	0.003
Translationally controlled tumour protein	gi|4507669	19697	4.84	632	61%	EIADGLCLEVEGK	716.944	2+	101	8.5E-08
						VKPFMTGAAEQIK	718.400	2+	50	0.0098
						EDGVTPYMIFFK	732.017	2+	48	0.016
						IREIADGLCLEVEGK	851.622	2+	111	6.5E-09
						DLISHDEMFSDIYK	857.088	2+	99	1.1E-07
						NYQFFIGENMNPDGMVALLDYR	1320.129	2+	110	6.9E-09

Protein	Accession[1]	Mr (calc)[2]	pI (calc)[2]	Protein score[3]	Coverage[4]	Peptide sequence[5]	m/z[6]	Charge	Ion score[7]	Expect[8]
Myotrophin	gi|21956645	13058	5.27	100	16%	GPDGLTAFEATDNQAIK	874.561	2+	92	6.3E-07
Heat shock protein 60	gi|306890	61157	5.70	155	6%	TALLDAAGVASLLTTAEVVVTEIPK	828.249	3+	101	4.4E-08
						TALLDAAGVASLLTTAEVVVTEIPKEEK	956.935	3+	51	0.0037
Proteasome alpha 5 subunit	gi|7106387	26565	4.74	726	63%	GVNTFSPEGR	532.332	2+	63	0.00061
						EELEEVIKDI	609.001	2+	57	0.0022
						LFQVEYAIEAIK	712.585	2+	91	6.9E-07
						ITSPLMEPSSIEK	724.481	2+	69	0.00013
						LGSTAIGIQTSEGVCLAVEK	1017.166	2+	93	3.0E-07
						IVEIDAHIGCAMSGLIADAK	700.828	3+	82	3.8E-06
						LNATNIELATVQPGQNFHMFTK	825.547	3+	77	1.2E-05
Nm23 protein (nucleoside diphosphate kinase A)	gi|35068	20740	7.07	407	55%	GLVGEIIKR	492.833	2+	66	0.00025
						GDFCIQVGR	526.363	2+	50	0.012
						NIIHGSDSVESAEK	743.470	2+	73	4.7E-05
Galectin-4	gi|5453712	36032	9.21	72	6%	FFVNFVVGQDPGSDVAFHFNPR	833.196	3+	72	2.9E-05
Aldehyde dehydrogenase 1A1	gi|21361176	55454	6.30	1055	41%	VAFTGSTEVGK	548.424	2+	60	0.0011
						QAFQIGSPWR	595.485	2+	64	0.00041
						LLLATMESMNGGK	698.980	2+	66	0.00024
						EEIFGPVQQIMK	718.063	2+	64	0.00037
						TIPIDGNFFTYTR	773.111	2+	87	1.7E-06
						ANNTFYGLSAGVFTK	795.629	2+	90	1.0E-06
						IFINNEWHDSVSGK	823.610	2+	83	4.4E-06
						IFVEESIYDEFVR	823.618	2+	71	7.4E-05
						ELGEYGFHEYTEVK	851.071	2+	54	0.0031
						LYSNAYLNDLAGCIK	858.127	2+	91	5.9E-07
						IAKEEIFGPVQQIMK	583.133	3+	47	0.021
						GYFVQPTVFSNVTDEMR	1003.740	2+	92	4.6E-07
						YILGNPLTPGVTQGPQIDK	671.212	3+	68	0.00012

Protein	Accession[1]	Mr (calc)[2]	pI (calc)[2]	Protein score[3]	Coverage[4]	Peptide sequence[5]	m/z[6]	Charge	Ion score[7]	Expect[8]
Lysosomal pepstatin insensitive protease	gi|2408232	61704	5.97	249	10%	LYQQHGAGLFDVTR	535.669	3+	80	8.8E-06
						AYPDVAALSDGYWVVSNR	992.177	2+	92	4.2E-07
						VPIPWVSGTSASTPVFGGILSLINEHR	945.659	3+	77	8.6E-06
Transgelin	gi|48255905	22653	8.87	467	56%	AAEDYGVIK	483.274	2+	66	0.0003
						LGFQVWLK	495.865	2+	59	0.0016
						TLMALGSLAVTK	610.957	2+	68	0.0002
						EFTESQLQEGK	648.373	2+	82	5.8E-06
						LVEWIIVQCGPDVGRPDR	703.816	3+	65	0.00023
						TDMFQTVDLFEGKDMAAVQR	778.774	3+	61	0.00047
Ornithine aminotransferase	gi|1168056	48847	6.39	471	30%	LPSDVVTAVR	528.855	2+	74	4.3E-05
						IVFAAGNFWGR	619.441	2+	51	0.0072
						TVQGPPTSDDIFER	781.466	2+	67	0.00017
						HQVLFIADEIQTGLAR	604.458	3+	50	0.0074
						AFYNNVLGEYEEYITK	977.068	2+	61	0.00058
						VAIAALEVLEEENLAENADK	714.118	3+	83	3.7E-06
Tropomyosin 4 isoform 1	gi|223555975	32874	4.69	143	7%	LVILEGELER	585.908	2+	60	0.0011
						IQLVEEELDR	622.399	2+	57	0.0026

Footnotes

[1]Accession numbers are from the NCBInr database

[2]Mr and pI are calculated from the protein sequence of the database entry

[3]Protein score refers to the standard Mascot protein score

[4]Coverage is the proportion of the protein sequence accounted for by all the matched peptides (including those with ions scores below the identity threshold)

[5]Data shown only for peptide matches with Mascot ions scores above the identity threshold (expect value < 0.05).

[6]m/z is the mass-to-charge ratio of the observed (peptide) ion

[7]Ion Score refers to the Mascot individual ion score

[8]Expect is the probability that the peptide match occurs by random chance
