# Supplementary material for: The Proteomics of Colorectal Cancer: Identification of a Protein Signature Associated with Prognosis
Source: PLoS One. 2011 Nov 18;6(11):e27718. doi: 10.1371/journal.pone.0027718 (PMC3220687; doi:10.1371/journal.pone.0027718)
Supplement: Table S4 — Relationship of survival of individual clusters identified by cluster analysis. (PDF) [file pone.0027718.s004.pdf]

Table S4. Relationship of survival of individual clusters identified by cluster analysis.

			**Cluster number**																			
			**1**		**2**		**3**		**4**		**5**		**6**		**7**		**8**		**9**		**10**	
**Cluster number (number of cases)**	**Number (%) of events**	**Mean survival (months, 95% CI)**	χ^2	**p value**	χ^2	**p value**	χ^2	**p value**	χ^2	**p value**	χ^2	**p value**	χ^2	**p value**	χ^2	**p value**	χ^2	**p value**	χ^2	**p value**	χ^2	**p value**
1 (39)	8 (20.5)	159 (135-178)			7.19	**0.008**	5.74	**0.017**	3.95	**0.047**	7.79	**0.005**	1.69	0.193	4.49	**0.034**	7.51	**0.006**	3.72	**0.054**	10.26	**0.001**
2 (24)	14 (59.3)	95(65-125)	7.13	**0.008**			0.62	0.430	0.15	0.699	0.12	0.734	0.53	0.466	0.39	0.531	0.05	0.821	1.04	0.308	1.97	0.160
3 (78)	36 (47.2)	105 (89-122)	5.74	**0.017**	0.62	0.430			0.00	0.986	0.20	0.657	0.08	0.772	0.01	0.944	0.80	0.370	0.12	0.732	2.61	0.106
4 (23)	10 (43.5)	100 (70-131)	3.95	**0.047**	0.15	0.699	0.00	0.986			0.06	0.806	0.09	0.771	0.00	0.988	0.33	0.566	0.10	0.748	1.75	0.186
5 (108)	51 (47.2)	98 (84-112)	7.79	**0.005**	0.12	0.734	0.20	0.657	0.06	0.806			0.18	0.670	0.04	0.840	0.31	0.575	0.62	0.431	2.08	0.149
6 (12)	5 (41.7)	88 (63-112)	1.69	0.193	0.53	0.466	0.08	0.772	0.09	0.771	0.18	0.670			0.04	0.836	0.76	0.382	0.00	0.998	1.86	0.173
7 (36)	16 (44.4)	93 (73-113)	4.49	**0.034**	0.39	0.531	0.01	0.944	0.00	0.988	0.04	0.840	0.04	0.836			0.57	0.468	0.14	0.704	1.76	0.184
8 (41)	19 (47.3)	89 (63-115)	7.51	**0.006**	0.05	0.821	0.80	0.370	0.33	0.566	0.31	0.575	0.76	0.382	0.57	0.468			1.26	0.263	0.74	0.389
9 (51)	21 (41.2)	103 (85-121)	3.72	**0.054**	1.04	0.308	0.12	0.732	0.10	0.748	0.62	0.431	0.00	0.998	0.14	0.704	1.26	0.263			3.06	0.080
10 (19)	11 (58.9)	56 (35-76)	10.26	**0.001**	1.97	0.160	2.61	0.106	1.75	0.186	2.08	0.149	1.86	0.173	1.76	0.184	0.74	0.389	3.06	0.080		

Significant values are highlighted in bold.
